# Comprehensive molecular pathology analysis of small bowel adenocarcinoma reveals novel targets with potential for clinical utility

**DOI:** 10.18632/oncotarget.4576

**Published:** 2015-07-30

**Authors:** Muhammad A. Alvi, Darragh G. McArt, Paul Kelly, Marc-Aurel Fuchs, Matthew Alderdice, Clare M. McCabe, Victoria Bingham, Claire McGready, Shailesh Tripathi, Frank Emmert-Streib, Maurice B. Loughrey, Stephen McQuaid, Perry Maxwell, Peter W. Hamilton, Richard Turkington, Jacqueline A. James, Richard H. Wilson, Manuel Salto-Tellez

**Affiliations:** ^1^ Northern Ireland Molecular Pathology Laboratory, Centre for Cancer Research and Cell Biology, Queen's University Belfast, Belfast, Northern Ireland, UK; ^2^ Tissue Pathology, Belfast Health and Social Care Trust, Belfast, Northern Ireland, UK; ^3^ Computational Biology and Machine Learning Laboratory, Centre for Cancer Research and Cell Biology, Queen's University Belfast, Belfast, Northern Ireland, UK; ^4^ Centre for Cancer Research and Cell Biology, Queen's University Belfast, Belfast, Northern Ireland, UK

**Keywords:** small intestine cancer, p53, Kazald1, CHN2, Pathology Section

## Abstract

Small bowel accounts for only 0.5% of cancer cases in the US but incidence rates have been rising at 2.4% per year over the past decade. One-third of these are adenocarcinomas but little is known about their molecular pathology and no molecular markers are available for clinical use.

Using a retrospective 28 patient matched normal-tumor cohort, next-generation sequencing, gene expression arrays and CpG methylation arrays were used for molecular profiling.

Next-generation sequencing identified novel mutations in *IDH1, CDH1, KIT, FGFR2, FLT3, NPM1, PTEN, MET, AKT1, RET, NOTCH1* and *ERBB4*. Array data revealed 17% of CpGs and 5% of RNA transcripts assayed to be differentially methylated and expressed respectively (*p* < 0.01). Merging gene expression and DNA methylation data revealed *CHN2* as consistently hypermethylated and downregulated in this disease (Spearman −0.71, *p* < 0.001). Mutations in *TP53* which were found in more than half of the cohort (15/28) and *Kazald1* hypomethylation were both were indicative of poor survival (*p* = 0.03, HR = 3.2 and *p* = 0.01, HR = 4.9 respectively).

By integrating high-throughput mutational, gene expression and DNA methylation data, this study reveals for the first time the distinct molecular profile of small bowel adenocarcinoma and highlights potential clinically exploitable markers.

## INTRODUCTION

The small bowel constitutes 80% of the length and 99% of the absorptive area of the gastrointestinal (GI) tract [1]. Despite this, tumors of the small bowel are rare, accounting for only 5% of all GI tract malignancies and are 50 times less common than large bowel tumors [1, 2]. Incidence rates however have been on the rise at a rate of 2.4% per year for the past ten years. There will be an estimated ∼9000 cases (a third of which will be adenocarcinomas) diagnosed this year in the US [3]. Risk factors include Crohn's disease, coeliac disease, Lynch syndrome, familial adenomatous polyposis (FAP) and Peutz-Jeghers syndrome (PJS), many of which are also shared by large bowel cancers [4].

Improvements in imaging and endoscopic techniques have led to improved detection of small bowel tumors. However, most small bowel adenocarcinomas (SBA) are still diagnosed at an advanced stage. Five year survival rates vary from 55% for stage I tumors to a dismal 5% for stage IV tumors [5]. Management of SBA also remains challenging and the role and type of adjuvant chemotherapy is not well defined at present [6, 7]. With current treatments there has been no change in survival rates over the last 20 years [8].

Compared to colorectal and gastric cancers our knowledge of the molecular pathology of SBA is limited and so there is a relative dearth of diagnostic, predictive and prognostic biomarkers. This has meant that the goal of personalized medicine for the treatment of SBA is yet to be achieved. *KRAS* and *TP53* mutations have been reported along with a low frequency of Her2 expression and *BRAF V600E* mutations [9]. Although SBA shares some histomorphological and molecular features with colorectal cancer, differences exist. For example, *APC* mutations are reported at a much lower frequency in SBA (5%) compared to colorectal cancer (80%) [1]. Aberrant expression of β-catenin, *SMAD4* mutations and loss of *DCC* are also reported at a lower frequency to that of colorectal cancer [1, 2, 9]. Accordingly, it is clear that data derived from the study of colorectal cancer cannot be extrapolated to inform management decisions for SBA.

To date most studies on the molecular characteristics in SBA have used a candidate based approach examining known biomarkers in other malignancies. The aim of this study was to perform a comprehensive high throughput analysis of the genetic, epigenetic and transcriptomic alterations that occur in this disease using a cohort of surgically resected cases in our institution. This has allowed us to identify novel candidate genes and molecular pathways that may have a significant role in the pathogenesis of SBA.

## RESULTS

### Next-generation sequencing

**Figure 1:**
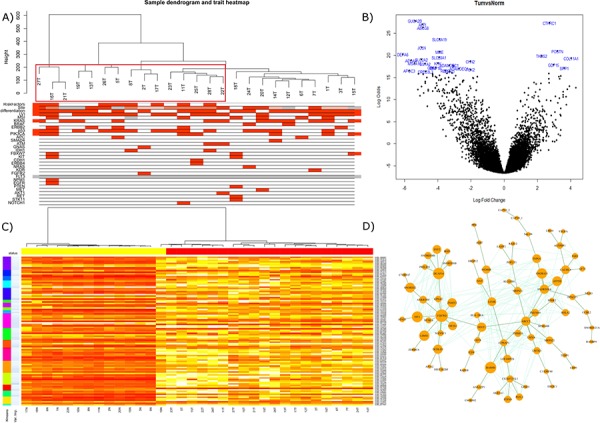
**A.** Gene expression of the tumor top 25% most variant probes overlain with associated clinical and mutational information using WGCNA. Clusters containing Crohn's and coeliac disease cases are outlined in red. **B.** Limma volcano plot of differential expression (non-variance filtered) with the top 30 probes annotated. **C.** Random forest classification on 75% of samples utilizing kmeans to offer data reduction of probe numbers for utility in validation machines of lower complexity. Top ranked variable importance in each kmeans group used in final *n* = 20 signature. **D.** WGCNA analysis revealed module ‘5′ had a strong correlation with *PIK3CA* mutation by Kendall correlation. Depicted here is the most strongly correlated network within the module, filtered across a bonferroni corrected all-possible-correlations threshold of *p* < 0.001 and visualized by NetBioV.

**Figure 2:**
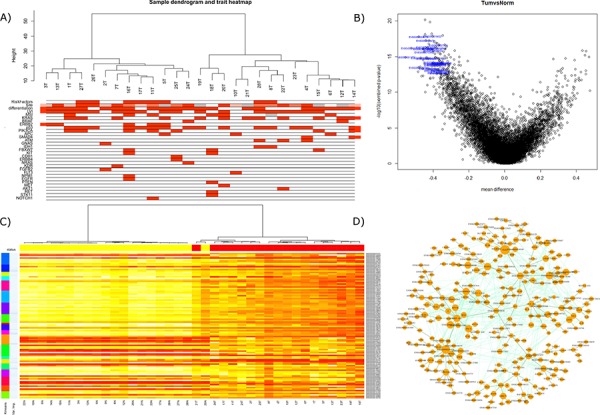
**A.** ‘Gene’ region methylation expression of the top 25% most variant probes overlain with associated clinical and mutational information. **B.** Volcano plot of differential expression (non-variance filtered) with the top 30 probes annotated, ranked by ‘combined rank’ in RnBeads. **C.** random forest classification on 75% of samples ‘gene’ regions utilizing kmeans to offer data reduction of numbers for utility in validation machines of lower complexity. Top ranked variable importance in each kmeans group used in final *n* = 20 signature. **D.** WGCNA analysis revealed module ‘3’ had a strong correlation with KRAS mutation by Kendall correlation. Depicted here is the most strongly correlated network within the module, filtered across a bonferroni corrected all-possible-correlations threshold of *p* < 0.001 and visualized by NetBioV.

We detected by our pipeline previously unknown mutations in SBA namely *IDH1*, *CDH1*, *KIT*, *FGFR2*, *FLT3*, *NPM1*, *PTEN*, *MET*, *AKT1*, *RET*, *NOTCH1* and *ERBB4* (Supplementary Table S2, NGS data can be accessed online at NCBI-SRA accession: PRJNA261313). The frequency of mutations in *TP53* and *KRAS* observed in our cohort were similar to those described previously [10]. Along with clinical and pathological data, we also matched mutational information against variance filtered gene expression and methylation data in Figure 1A and 2A. Our next-generation sequencing approach has been validated on hotspot mutations in *KRAS*, *BRAF*, *TP53*, *ERBB2*, *IDH1* and *KIT* with Sanger sequencing assays (data not supplied).

*TP53* mutational information conferred a significant survival advantage for *TP53* wild-type patients, *p* = 0.0345 and HR = 3.2 (Figure 4A). We did not see survival advantage in MSI, *PIK3CA*, *KRAS* or any other stratified groups (Figures not supplied).

### Gene expression

Good quality data was obtained from 20 normals and 25 tumors and can be accessed online at NCBI GEO accession GSE61465 (http://www.ncbi.nlm.nih.gov/geo/query/acc.cgi?token=gtqzsscmdlkjfqn&acc=GSE61465). A list of the most differentially expressed genes between tumor and normal are supplied in Supplementary Table S3A. The volcano plot depicted in Figure 1B, annotates the top 30 most differentially expressed probes. The top 25% of the most variable probes as hierarchically clustered by flashClust and with mutational/trait overlay describes a variable dataset in the tumor only samples, which may have an (auto)immune component due to the Crohn's and Coeliac disease background of these cases (enclosed in red, Figure 1A). In order to generate a classification of tumor versus normal; a random forest signature (*n* = 20) was able to classify the training set with only one false positive, also evidenced in the MDS plot (Supplementary Figure S4A). Applying this signature to our test set (*n* = 11), we were able to call all cases (5 ‘tumors’ and 6 ‘normals’) accurately (Supplementary Table S2G). The signature of the top 100 gene list clustered by kmeans and ranked by variable importance is depicted in Figure 1C. WGCNA analysis identified 10 modules for the gene expression data, where we focused on module 5 which was strongly correlated with *PIK3CA* (*p* = 0.04) of which, the largest connected complex is depicted by the NetBioV R package in Figure 1D (for other modules and final data used in trait relationship see Supplementary Figure S1, Supplementary Figure S3IIA and SIIIA). DAVID functional annotation clustering mapped 75IDs of the 79IDs and revealed 27 clusters which can be found in Supplementary Table S2I.

### DNA methylation

According to RnBeads; hypermethylation was found to be much more prevalent than hypomethylation (11% vs 6%, *p* < 0.01). The remaining 83% of probes remained unaffected. CpG sites associated with genes bodies, promoters and CpG islands all exhibited higher levels of hypermethylation compared to hypomethylation (12% vs 7%, 20% vs 1% and 12% vs 6% respectively *p* < 0.01). DNA methylation trends are detailed in Supplementary Table S3b–S3f and array data can be accessed online at NCBI GEO accession GSE61467 (http://www.ncbi.nlm.nih.gov/geo/query/acc.cgi?token=sbqdmggqtjsrbqj&acc=GSE61467).

Good quality data was obtained for all samples. RnBeads analysis of the raw idat files allowed us to analyze the differential methylation across normal vs tumor and across tumor only stratifying by mutations. The differential ‘gene’ region methylation is depicted in Figure 2B: volcano plot, with the top 30 genes, by combined rank, annotated by their Ensembl ids (Lists of differentially methylated analyses are supplied in Supplementary Table S3B to S3F). The WGCNA flashClust hierarchical clustering of the tumor gene regions depicts two groupings, with underlying clinical and mutational information (Figure 2A). These groupings were also somewhat conserved in the RnBeads PCA plots, but with a strong separation between tumor and normal (Supplementary Figure S4B for sites, Supplementary Figure S4C for genes and 4D for promoters), and fluctuate slightly when examined by RnBeads top 1000 most variant genomic regions’ heat map (CpG islands, tilling and promoters) and across sites (Figures not supplied). WGCNA network analysis identified 6 modules for the gene region methylation data of which module 3 became a focus as it was found to have a strong correlative trend with *KRAS* mutation status (0.1 with Kendall correlation, originally significant with Pearson, for other modules and final data used in trait relationship see Supplementary Figure S2, Supplementary Figure S3IIB and SIIIB). DAVID functional annotation clustering mapped 172 IDs of the 265 in module 3′s largest complex and revealed 80 clusters which can be found in Supplementary Table S3J.

Finally, we analyzed the gene regions of the methylation data by random forest to extract a signature that could best encapsulate tumor vs normal with a minimal classification error. Following the same protocol as gene expression and described below, the 20 gene signature was able to classify all tumors accurately with one normal and one tumor misclassified (The 100 gene signature is depicted in Figure 2D, 20 gene performance and list is given in Supplementary Table S3G). Applying this to the test set (*n* = 17) the signature was able to accurately predict the disease type of all samples (8 normals, 9 tumors). Here we also found a candidate of interest; *Kazald1*, which not only demonstrated utility in the robust classifier to partition tumor versus normal samples, but within tumor variation, when stratified on the median methylation proffered prognostic significance (*p* = 0.0079, HR = 4.9), with worse prognosis for lower methylation (see Figure 4B).

### DNA methylation and gene expression correlation

**Figure 3:**
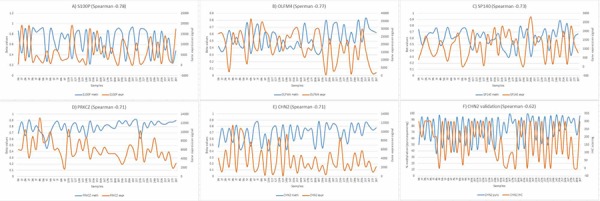
Correlation between DNA methylation and gene expression **A–E.** Inverse correlation for the top 5 genes from array data, *n* = 45 (primary axis – methylation, secondary axis – expression). **F.** Inverse correlation between pyrosequencing and immunohistochemistry for *CHN2*, *n* = 56.

GenomeStudio was used to correlate gene expression with DNA methylation and targets were ranked according to the Spearman correlation coefficient. There were 486 combinations (for 266 unique transcripts) with a Spearman correlation coefficient ≤ −0.5 with 70% hypermethylated (downregulated) and 30% hypomethylated (upregulated) in tumor vs normal. Data for the top 5 genes are plotted in Figure 3A–3E. With 9 methylation array probes all correlating with two gene expression probes below a Spearman correlation coefficient of −0.5, *CHN2* was selected for validation. Validation for DNA methylation was done using pyrosequencing and gene expression using immunohistochemistry (see Figure 3F).

**Figure 4:**
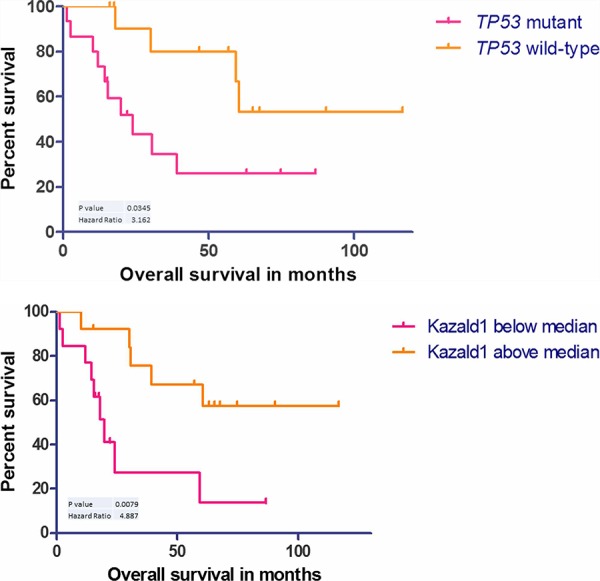
**A.** Kaplan–Meier curve showing survival advantage for wild-type *p53* cases, *n* = 27. **B.** Kaplan–Meier curve showing survival disadvantage for cases with *Kazald1* hypomethylation, *n* = 26.

### MSI

Six out of the 28 cases (∼20%) showed high MSI defined as two or more markers affected.

### Immunohistochemistry

**Figure 5:**
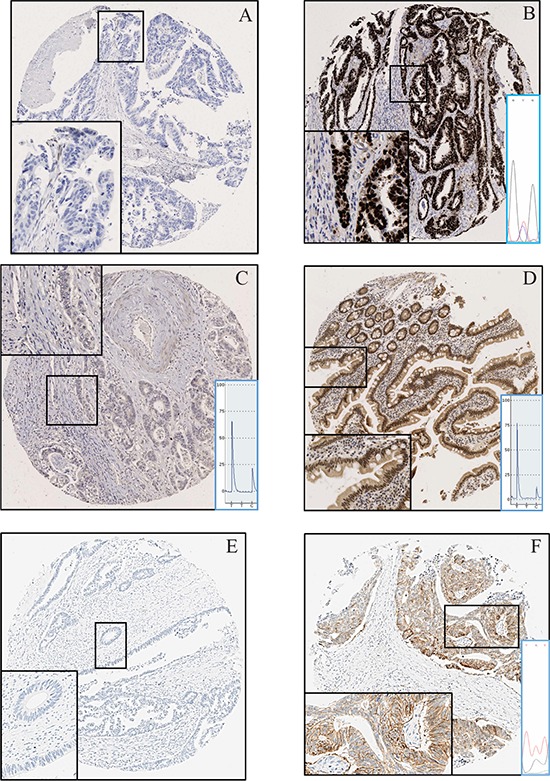
Immunohistochemistry **A.** *p53* wild type tumor showing no staining (extreme negative). **B.** *p53* mutant tumor showing extreme positive staining. The C > T substitution can be seen as confirmed by Sanger sequencing **C.** No CHN2 protein expression in tumor. A low T and high C peak show a highly methylated CpG **D.** CHN2 protein expression in normal small bowel. A high T and low C peak show an unmethylated CpG **E.** Tumor with no *Her2* expression. **F.** Tumor with a *Her2* c.2329G > T (Substitution) mutation expressing the protein.

p53 extreme positive staining as shown in Figure 5B was observed in nine cases (32%) [11]. All these cases were also mutant for *TP53*. Extreme negative staining is shown in Figure 5A. Scoring for CHN2 was done using the formula ‘percent area of core staining positive X intensity of staining (ranging from 0–3)’. A representative tumor and normal core can be seen in Figure 5C and 5D respectively and as shown in Figure 3F, tumors consistently scored lower than normals. Based on the recommendations by Wolff *et al*., 2013, only three cases showed Her2 positivity (Figure 5F). For comparison a representative Her2 negative core can be seen in Figure 5E [12].

## DISCUSSION

The present NGS analysis has confirmed previously reported mutations and their respective frequencies in SBA [10], and coupled with this we report novel mutations in multiple genes; namely *IDH1*, *CDH1*, *KIT*, *FGFR2*, *FLT3*, *NPM1*, *PTEN*, *MET*, *AKT1*, *RET*, *NOTCH1* and *ERBB4* (Figure 1A and 2A show mutations in relations to gene expression and DNA methylation). On average, patients in our cohort demonstrated 2.6 mutations with eight patients having only one mutation to a single patient having seven.

*TP53* mutations were found in over 50% of the sample cohort (15/28 cases). This is concordant with the ∼40% frequency reported previously [10, 13], when relative study sizes are considered. Apart from two cases which harbored mutations in exon 10, most *TP53* mutations were identified in exons 5 to 8 (exon 5 – 2 cases (14%), exon 6 – 1 case (7%), exon 7 – 4 cases (29%) and exon 8 – 7 cases (50%)). One case had three *TP53* mutations: two in exon 8 and one in exon 10. As shown previously, *TP53* mutations were also indicative of poor survival in our cohort (Figure 4A) [10]. *KRAS* mutations were found in 12 cases (42%) out of which 9 (32%) were in exons 12/13. This is also consistent with the ∼40% frequency reported by Rashid *et al*., 1997 and Aparicio *et al*., 2013 [9, 13]. The three remaining cases harbored a mutation in codon 14, 117 and 146. To our knowledge mutations in codon 14 and 117 have not been reported previously in SBA. c.351A > C (substitution) in codon 117 just like c.436G > C (Substitution) has therapeutic implications in colorectal cancer and is known confer reduced sensitivity to anti-EGFR antibodies like cetuximab/panitumumab [14]. *APC* mutations which are characteristic of colorectal cancer were only found in two patients. Also there was one additional case with a novel mutation (c.4744G > C (substitution - missense) aa1582 alanine to proline – exon 15). We also observed mutations in *ERBB2* not reported previously in SBA (Supplementary Table S1). Out of these we found c.2329G > T (Substitution - Missense p.V777L) to also correlate with Her2 expression (Figure 5F). We also found two novel mutations XM_005257139.1:c.2476C > G (leucine to valine) and M_005257139.1:c.2272G > A (valine to methionine). Three cases (∼10%) were positive for Her2 expression, which in theory can benefit from anti-*ERBB2* therapy and is similar to what has been reported previously [10].

Only one patient had a *BRAF V600E* mutation which is in line with the low frequency of *BRAF* mutations reported previously in SBA [9, 15]. However there were another two cases; one with a mutation in exon 15 (alongside a mutant *KRAS*) the other in exon 11. All three mutations however lie in the kinase domain where they are known to be activating and potentially transforming [16]. Mutations in *BRAF* are known to negatively impact anti-EGFR therapy in colorectal cancer but have a beneficial effect in melanoma in response to the use of BRAF/MEK inhibitors [17, 18]. *IDH1* mutations, which are commonly observed in gliomas and leukemias, were found in two cases of our cohort. This may have therapeutic consequences in SBA as recently it has been shown that mutant *IDH1* can be targeted with anti-tumor vaccines [19].

To our knowledge gene expression and DNA methylation changes have never been studied in SBA. From the gene expression data almost 5% of the ∼25000 RNA transcripts were observed to be dysregulated. Of these 40% were upregulated and 60% downregulated in tumor tissue compared to normal (Supplementary Table S3A). We also observed that cases in which there was a history of Crohn's and coeliac disease depicted greater dissimilarities and distances in the dendogram when tumors were clustered based on the most variable probes (Figure 1A). From the methylation arrays our study found 17% of the CpGs assayed to show statistically significant changes in methylation between normal and tumor tissue (Supplementary Table S3B). This is equal to 68, 592 individual CpGs. No changes in methylation were observed for *MLH1* or other CIMP genes as reported previously [15]. Our analysis returned 266 genes (RNA transcripts) which had an inverse correlation between expression and methylation on an associated CpG. One of the top ranked candidates, *CHN2*, was selected for further validation. It has been linked to progression in malignant gliomas and has also been shown to be downregulated in breast cancer and breast cancer cell lines. Restoring CHN2 expression in MCF7 cell line using adenoviral delivery leads to cell cycle arrest and an inhibition of proliferation [20] [21]. Our analysis demonstrates that CHN2 expression in SBA may be controlled by DNA methylation as DNA hypermethylation accompanies its downregulation. As this trend was observed in all normal-tumor pairs, *CHN2* methylation has potential to act as a biomarker for SBA screening in blood/stool samples.

Data classification by random forest methodology allowed us to implement a process to generate succinct lists that can best characterize tumor against normal in the expression data [22]. The 100 gene lists (Figure 1C and 2C) depict the utility of the test classifier to separate tumor vs normal samples with kmeans (*k* = 20), partitioning the lists into 20 clusters ranked by their variable importance. *k* = 20 was chosen to allow for succinct lists that could be used in validation platforms with lower complexity as well as proffering succinct divergent candidates that could become potential biomarkers. *CHN2* is one such candidate that performed well in *n* = 100 gene expression classifier (ID: ILMN_2403237) and was retained in the robust *n* = 20 list. In the methylation data the candidate of interest Kazal-type serine peptidase inhibitor domain 1 (*Kazald1*) has been shown to demonstrate a shorter overall survival for patients with hypomethylation in gliomas and is suggested to promote progression through invasion and proliferation [23].

In the network analysis correlating the module eigengene's significance with sample traits (clinicopathological and mutational data) allowed us to measure the association of the modules-trait relationship. Significant associations of interest were Module 5′s eigengene correlation with *PIK3CA* (gene expression data) and *KRAS* (methylation ‘gene’ region data). The largest connected component of these modules was visualized using NetBioV to reveal insight into their connectivity (Figure 1D and 2D). Measuring gene significance with module membership allowed us to measure their central players. To validate their performance key genes were rechecked to a limma differential expression using *PIK3CA* (gene expression) and *KRAS* (methylation) mutational stratification (data not supplied). The key gene significance candidates to the specific modules were strongly evidenced in these lists supporting their strong differential as well as their inter-activity. DAVID functional analysis of the largest interconnected complex annotations are supplied in Supplementary Table S3I and S3J where the gene expression analysis in the top three DAVID clusters detail splicing, mRNA processing along with transmembrane transporter activity and GTPase binding. Of note, was cluster five depicting vasculature development and angiogenesis as key functions. The methylation top three DAVID clusters list, among other functions, homeobox, regulation of transcription and neuron development.

In summary, this study has for the first time highlighted the extent of molecular changes associated with SBA. Our data convergence study utilizing high-throughput technologies has elucidated key mutations, RNA and methylation drivers in SBA. The clinical potential of *TP53* mutations and *Kazald1* hypomethylation as prognostic biomarkers and *CHN2* as a diagnostic biomarker are focus areas for further research by our group.

## MATERIALS AND METHODS

### Patient clinical and pathological data

Ethical permission for the study was given by the Northern Ireland Biobank (Ethics: 11/NI/0013/NIB13–0067). Twenty-eight patients who underwent surgical resection of SBAs between 2002 and 2013 were identified from the pathology archives of the Belfast Health and Social Care Trust (BHSCT). Relevant patient demographics and clinical data were reviewed and recorded (summarized in Table 1).

**Table 1:** Patient demographics and clinical data

**No of patients**	28 - Collected b/w 2002–13
**Gender**	16 female (57%), 12 male (43%)
**Age**	Average 61y
	Range 32–85
**Tumor size**	Average 4.5 cm
	Range 2–12 cm
**Tumor location**	6 Duodenum
	2 DJ flexure
	8 Jejunum
	7 Ileum
	5 Small intestine NOS
**Differentiation**	7 poor (25%)
	21 moderate (75%)
**Risk factors**	8 (including both Crohn's and coeliac disease)
**T stage**	16 T4, 12 T3
**N stage**	6 N0, 17 N1, 2N2, 3Nx
**M Stage**	6M1, 21Mx, 1 unknown

Formalin-fixed, paraffin-embedded (FFPE) blocks and slides were obtained for each of the cases. A full histopathology review was undertaken by a GI pathologist (PK) to confirm that each of the cases represented primary SBA. Patients who had ampullary/periampullary adenocarcinomas were not included in the study cohort. The histopathological parameters relevant to staging and prognosis according to the American Joint Committee on Cancer (AJCC) were also reviewed and verified [24]. Representative tumor and normal blocks were selected from each case for further immunohistochemical and molecular analysis (see below).

### Nucleic acid extraction and TMA (tissue microarray) construction

Additional hematoxylin and eosin stained sections of normal and tumor were prepared from the selected FFPE blocks and annotated by a GI pathologist (PK) for DNA/RNA extraction and TMA construction. For both DNA and RNA extractions, 6 × 5 micron blank sections were cut from each block and dewaxed in xylene and alcohol. Under direct visualization using magnifying glass annotated areas were scrapped off using a scalpel blade into 1.5 ml tubes. Maxwell 16 FFPE Plus LEV DNA Purification Kit (Promega, UK) was used for DNA extraction and RNeasy FFPE Kit (Qiagen, UK) for RNA extraction. Elution was in a volume of 50 ul. TMAs were constructed using 1 mm cores from tumors in triplicate and normals in duplicate on a Beecher MTA1 (Beecher Instruments Inc., WI), following international standards [25].

### Next-generation sequencing

Next-generation sequencing was carried out according to manufacturer's instructions on the Ion PGM™ System using Ion AmpliSeq Cancer Hotspot Panel v2 (Life Technologies, UK). 50 ng of DNA was used. Libraries were prepared using Ion AmpliSeq Library Kit 2.0 and quantified using the Agilent 2100 Bioanalyzer system (Agilent Technologies, UK). The Ion OneTouch 2 System was used to generate template-positive sphere particles for sequencing. Normals were run once and tumors in duplicate on Ion 318 chips (Life Technologies, UK).

### Gene expression

Whole-Genome DASL HT Assay was used for gene-expression profiling according to manufacturer's instructions. 100ng of RNA was used. The resulting PCR products were hybridized onto the HumanHT-12 v4 BeadChip and scanned using iScan Microarray Scanner (Illumina Inc., UK).

### DNA methylation

The Infinium HumanMethylation450 BeadChip kit (Illumina Inc., UK) was used on 200 ng of DNA which was restored according to the manufacturer's instructions. EZ Methylation Kit and ZR-96 DNA Clean & Concentrator-5 (Zymo Reseach, CA) were used for bisulfite conversion. Chips were scanned using iScan Microarray Scanner.

### Sanger sequencing

Sanger sequencing was carried out using BigDye Terminator v3.1 Cycle Sequencing Kit on ABI 3500XL genetic analyzer using manufacturer's instructions. Primers were either obtained from the Northern Ireland Biobank or designed using NCBI primer design tool with M13 overhangs (Supplementary Table S1). PCR was carried out using AmpliTaq Gold 360 Master Mix (Applied Biosystems, UK) and clean-up using ExoSAP-IT (Affymetrix, UK). All mutations were confirmed manually using Finch TV version 1.4.0 (Geospiza Inc., WA).

### Pyrosequencing

Pyrosequencing assays were designed using PyroMark assay design software v2.0.1.15 (Supplementary Table S1) and run with PyroMark Q24 v2.0.5 software (Qiagen, UK) on a PyroMark Q24 (PyroMark, Sweden) according to manufacturer's instructions. 200ng of DNA was bi-sulfite converted using EZ DNA Methylation Kit (Zymo Research, USA) into an elution volume of 30ul. PCR was performed using ImmoMix (Bioline, UK).

### Immunohistochemistry

3 micron TMA sections were used for all immunohistochemistry (IHC). CHN2 and p53 IHC was carried out on a fully automated Leica BOND-MAX (Leica Microsystems, UK). Anti-CHN2 HPA018989-100UL (Sigma, UK) was used at a 1:25 dilution. Heat assisted antigen retrieval was used for 20 min. Anti-p53 M7001 (Dako, UK) was used at a 1:100 dilution and heat assisted antigen retrieval for 30 min. ERBB2 IHC was performed on a Ventana Benchmark XT platform using ultraView Universal DAB Detection Kit and PATHWAY anti-HER-2/neu (4B5) Rabbit Monoclonal Primary Antibody (Ventana Medical Systems, UK). Antigen retrieval was with Cell Conditioning 1 solution for 16 min.

### Microsatellite instability analysis

MSI analysis was performed on a MSI Analysis System, Version 1.2 (Promega, UK) according to manufacturer's instructions. The five mononucleotide repeat markers tested (BAT-25, BAT-26, NR-21, NR-24 and MONO-27) were co-amplified using fluorescently labelled primers and analyzed on an ABI 3500XL genetic analyzer.

### Data analysis

#### Next-generation sequencing

Data analysis was carried out in tandem between CLC genomics Workbench 6.5 (CLC GW) and confirmed with Ion Torrent's variantCaller (v4.0-r76860) using default selection commands. These were read into CLC using the default clipping and trimming functions. Calls identified from CLC GW were sorted by coverage (>20) and frequency (>5%) and verified with variantCaller.

#### Gene expression

Data were read using the limma package for R statistical environment [26, 27]. Background correction followed by quantile normalization was performed using limma's neqc function and control probe information, and the intensities were log2 transformed. This was followed by an unsupervised hierarchical clustering analysis using pvclust [28] and resultant heatmaps were generated by heatmap.plus [29]. Differential expression was also performed using the limma package. Probes expressed in at least one quarter of the arrays to a detection *p*-value of ≤ 0.05 were retained. Subsequent differential expression lists, MDS plots and volcano plots were generated through limma package functions.

#### DNA methylation

After initial QC using GenomeStudio, the raw idat files were utilized in the RnBeads software package for unsupervised analysis and differential methylation. The pipeline was run with the removal of sex linked sites followed by methylumi background correction and bmiq normalization [30, 31]. All samples were run in a pipeline depicting the tumor vs normal samples, and wild-type vs mutant in mutations of high frequency (*n* > 3). From here the tumor samples were extracted to create a tumor only RnBead set for downstream analysis.

#### Gene expression and DNA methylation

##### Random forest

Classification on normal vs tumor samples was performed on methylation gene region and gene expression filtered data using the package randomForest [32] where an approach similar to Griffith *et al*., 2013 was adopted [22]. The resultant output was utilized to derive a list of the top 100 by variable importance (Gini). kmeans clustering (*k* = 20) was used to alleviate data redundancy in the generated list. Top 100 list and top 20 analyzed are supplied in Figure 1C and 2C. (Random forest analysis: Supplementary Table S3G–S3H and 20 gene signature bootstrap: Supplementary Figure S3IA and SIB).

##### WGCNA (weighted gene correlation network analysis)

Tumor only data (gene regions for methylation) was first filtered by taking the top 25% most variant probes using genefilter's varFilter function. The Scale Free Topology plot was used either selecting the lowest power where the curve flattens out or the scale free R^2 > 0.8 (value equated to 6 in gene expression and 12 in methylation). Associations to traits/mutations were identified using the module eigengene's correlation with the external trait information, which were set stringently (Supplementary Figure S3 IIA and S IIB). Module visualization was performed using NetBioV where, a filter was applied on the distribution of all possible correlations among the modules by a bonferoni corrected *p*-value ≤ 0.001 [33]. The largest connected complex in each module was selected for NetBioV. All modules’ largest connected complexes not selected for main manuscript (Figure 1D and 2D) are supplied as supplementary data (Supplementary Figure S1 and S2), where any connected hubs above 10 vertices other than the main complex, denoted by the specific module plus ‘B’, ‘C’ etc. have also been included. DAVID functional annotation analysis was then used on the key largest connected complexes [34, 35].

#### Survival

Kaplan-Meier overall survival analysis was performed on the cohort defined as from the date of resection to the date of death or date last seen, and given in months. The *p*-value used is that of the log-rank test and analysis was performed using Graphpad Prism 5 v5.03. Gene expression or methylation values threshold was pre-defined to be dichotomized on the median value for survival purposes.

## SUPPLEMENTARY FIGURES AND TABLES






