# Supplementary material for: Comprehensive molecular pathology analysis of small bowel adenocarcinoma reveals novel targets with potential for clinical utility
Source: Oncotarget. 2015 Jul 30;6(25):20863–74. doi: 10.18632/oncotarget.4576 (PMC4673235; doi:10.18632/oncotarget.4576)
Supplement: Supplementary file 1 [file oncotarget-06-20863-s001.pdf]

SUPPLEMENTARY FIGURES AND TABLES

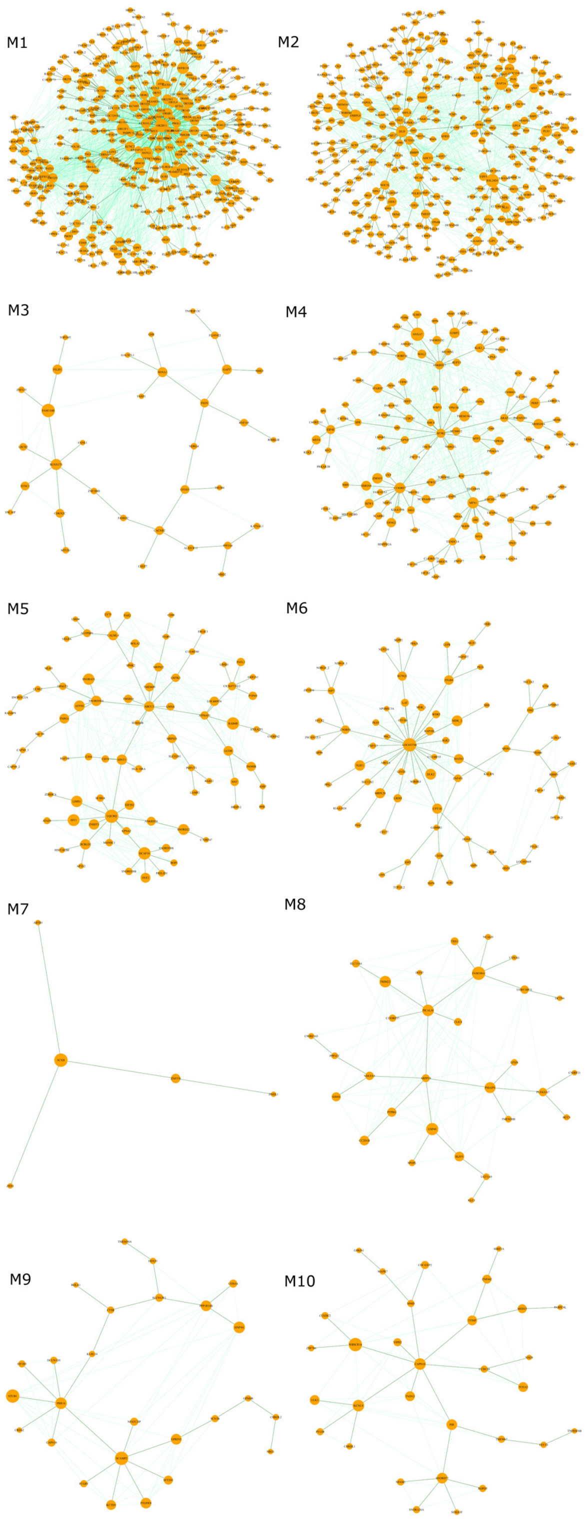

Supplementary Figure S1: Composite modules (Expression). Modules 1–10 of WGCNA analysis. Only large complexes were depicted that contained over 10 vertices in each module using the settings supplied in the methods section. These modules are visualized using NetBioV and annotated by the gene name.

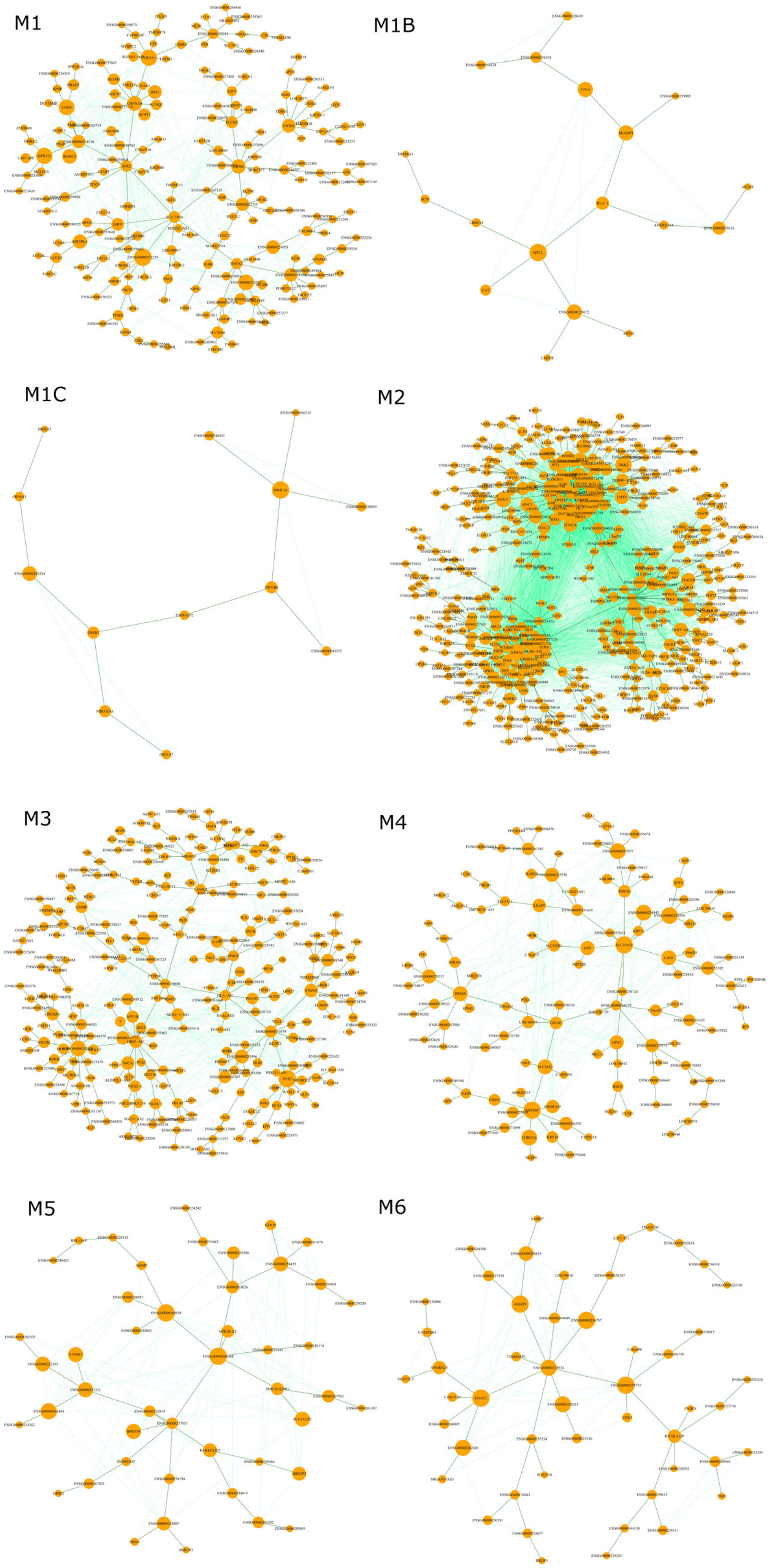

Supplementary Figure S2: Composite modules (Methylation). Modules 1–6 of WGCNA analysis, Module 1 had two extra, large connected complexes included in this figure (M1B and M1C) which satisfied our threshold of over 10 vertices, these are depicted by NetBioV software employing the minimum spanning tree algorithm.

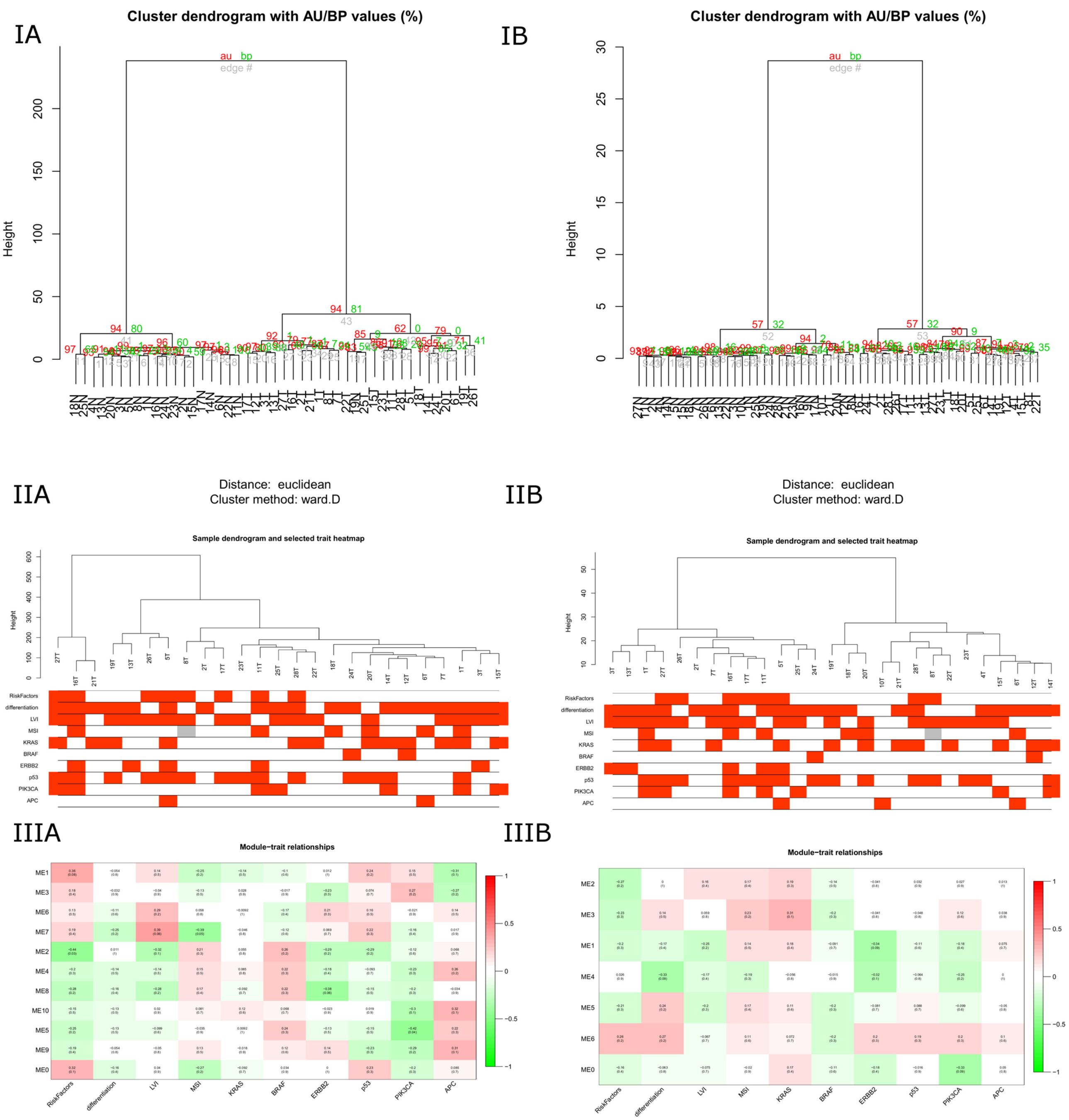

Supplementary Figure S3: Random forest signature of the *n* = 20 list to visualize the robust separation of tumor versus normal samples by bootstrap sampling using pvclust (IA gene expression and IB methylation), IIA and IIB depict the binary traits that were used in the WGCNA analysis to correlate with the module eigengenes and the resultant analysis by Kendall correlation relationship (IIIA gene expression and IIIB methylation) depicting module 5 eigengene (gene expression) correlated with *PIK3CA* and module 3 eigengene with a strong correlative trend with *KRAS* in the methylation gene region data.

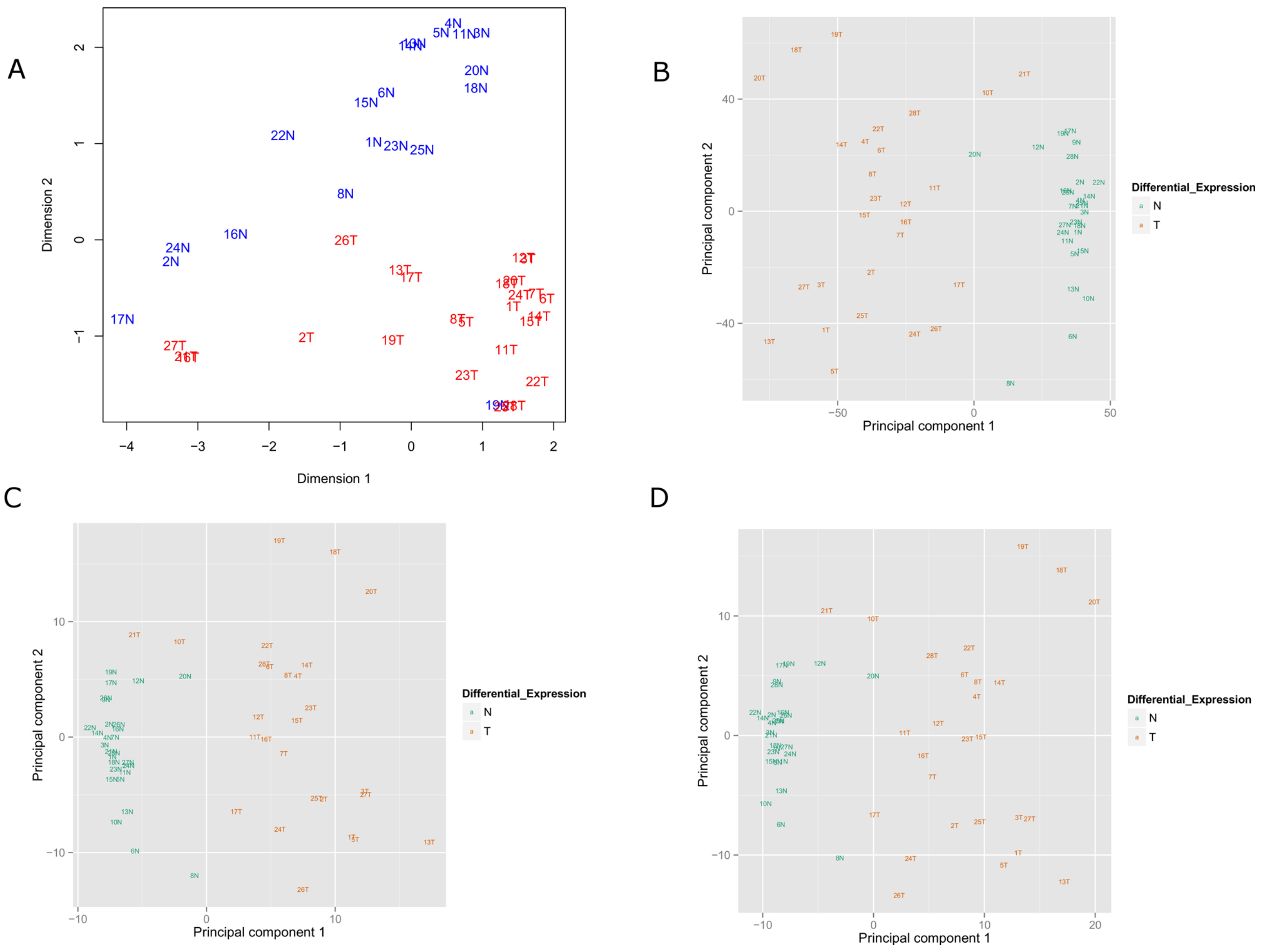

Supplementary Figure S4: A. MDS (limma) plot of the gene expression data which demonstrates our one outlier ’normal’ sample amongst the tumor groupings (19N). **B. C.** and **D.** PCA plots from the RnBeads software depicting the data patterns of tumor vs normal in ’sites’, ’genes’ and ’promoters’ respectively which was used in the pipeline for our differential expression using RnBeads.

Supplementary Table S1: Sanger sequencing and pyrosequencing primer sequences

Sanger sequencing

BRAF Fw v600	CTACTGTTTTCCTTTACTTACTACACCTCAGA
BRAF Rv v600	ATCCAGACAACTGTTCAAACTGATG
KRAS F c12-13-M13	aag gcc tgc tga aaa tga ctg
KRAS R c12-13-M13	aga atg gtc ctg cac cag taa
KRAS F c61-M13	GAA GTA AAA GGT GCA CTG TAA TAAT
KRAS R c61-M13	TTT AAA CCC ACC TAT AAT GGT
UNV Sequencing primer-F	GTTGTAAAACGACGGCCAGT
UNV Sequencing primer-R	CACAGGAAACAGCTATGACC
IDH1_F1	GTTGTAAAACGACGGCCAGTGCCAACATGACTTACTTGATCCC
IDH1_R1	CACAGGAAACAGCTATGACCGGCTTGTGAGTGGATGGGTA
KIT_F1	GTTGTAAAACGACGGCCAGTTCTGTTTTTCTTGGCAGGCTC
KIT_R1	CACAGGAAACAGCTATGACCTCCATTTGACAAAGCCCGGA
TP53_exon8_F1	GTTGTAAAACGACGGCCAGTTCCTTACTGCCTCTTGCTTCTC
TP53_exon8_R1	CACAGGAAACAGCTATGACCCTCCACCGCTTCTTGTCCTG
TP53_exon7_F1	GTTGTAAAACGACGGCCAGTTCTTGGGCCTGTGTTATCTCC
TP53_exon7_R1	CACAGGAAACAGCTATGACCAGAAATCGGTAAGAGGTGGGC
TP53_exon6_F1	GTTGTAAAACGACGGCCAGTGATTGCTCTTAGGTCTGGCCC
TP53_exon6_R1	CACAGGAAACAGCTATGACCAGACCCCAGTTGCAAACCAG
TP53_exon5_F1	GTTGTAAAACGACGGCCAGTCTACAGTACTCCCCTGCCCT
TP53_exon5_R1	CACAGGAAACAGCTATGACCCAGCTGCTCACCATCGCTA
ERBB2_c.2329-M13_F1	GTTGTAAAACGACGGCCAGTTGTGTGGTCTCCCATACCCT
ERBB2_c.2329-M13_R1	CACAGGAAACAGCTATGACCAGGGCATAAGCTGTGTCACC
ERBB2_c.2524-M13_F1	GTTGTAAAACGACGGCCAGTGGTCTACATGGGTGCTTCCC
ERBB2_c.2524-M13_R1	CACAGGAAACAGCTATGACCGTTGGGACTCTTGACCAGCA
Pyrosequencing	
CHN2_F1	TGTTGATAGATTGAGGATGTATTAGGAG
CHN2_R1	Biotin-AATTACATATTAAAAAAAAACATACTACAAAAAC
CHN2_S	TGATAGATTGAGGATGTATTA

Supplementary Table S2: Mutations per sample—detailed information including location, coverage, frequency etc

Supplementary Table S3A: Differential expression gene list ranked by Benjamini Hochberg False Discovery Rate

Supplementary Table S3B: Differential methylation gene list ranked by combined rank—Sites

Supplementary Table S3C: Differential methylation gene list ranked by combined rank—Tilling

Supplementary Table S3D: Differential methylation gene list ranked by combined rank—Genes

Supplementary Table S3E: Differential methylation gene list ranked by combined rank—Promoter

Supplementary Table S3F: Differential methylation gene list ranked by combined rank—Islands

Supplementary Table S3G: Classification random forest; 20 gene analysis (expression)

Supplementary Table S3H: Classification random forest; 20 `gene' region analysis (methylation)

Supplementary Table S3I: DAVID functional annotation analysis of gene expression Module 5's largest complex annotation list

Supplementary Table S3J: DAVID functional annotation analysis of methylation Module 3's Ensembl gene IDs from the largest connected complex
